# Glass ceiling among female prosthetists & orthotists: Perceptions, opportunities and strategies for moving forward

**DOI:** 10.33137/cpoj.v8i1.44720

**Published:** 2025-06-17

**Authors:** A Eshraghi, Z Safaeepour

**Affiliations:** 1. West Park Healthcare Centre, University Health Network, Toronto, Canada.; 2. Department of Human Performance and Health, University of South Carolina Upstate, Spartanburg, South Carolina, United States.

**Keywords:** Glass Ceiling, Prosthetics, Orthotics, Gender Inequality, Diversity, Prosthetists, Career Advancement, Leadership, Women, Gender, Orthotists

## Abstract

**BACKGROUND:**

The glass ceiling, a metaphorical barrier hindering women's career advancement, is prevalent across various sectors, including healthcare. Women have proved their competence as successful senior-level leaders. Despite this, there is still a striking under-representation of female prosthetists and orthotists in leadership positions as managers and business owners. This study investigated the “glass ceiling𠇜 phenomenon in the prosthetics and orthotics (P&O) field, where women, despite growing representation in the profession, are underrepresented in leadership roles.

**OBJECTIVE:**

This study aimed to examine the beliefs and expectations of female prosthetists and orthotists regarding career advancement and leadership opportunities.

**METHODOLOGY:**

This survey study had two sections; the first section was general demographic questions, and any gender could participate but the second section was the Career Pathways Survey (CPS), which assessed beliefs about the glass ceiling and only female practitioners could participate. The survey evaluated four factors: Denial, Resilience, Acceptance, and Resignation, to understand how women perceive their ability to break through the glass ceiling. All individuals with a professional qualification in prosthetics and/or orthotics were eligible to participate. The survey was distributed worldwide through the ISPO and other professional associations. The survey was opened in April 2021 and remained active for six months.

**FINDINGS:**

110 female participants completed the survey. The participants were mainly from North America, Europe and Australia. Findings revealed that factors like age, marital status, and salary were significant predictors of different belief scores, particularly with respect to career acceptance and denial. Results highlighted that women's beliefs about their career advancement were influenced by their personal life situations, such as having children, and the number of years of professional experience.

**CONCLUSION:**

The study calls for organizational reforms to address gender inequalities by implementing policies that support work-life balance, mentoring, and career development. It also emphasizes the importance of further research, particularly examining the intersectionality of gender, race, and other diversity factors, to provide a comprehensive understanding of barriers to leadership in P&O and other healthcare fields.

## INTRODUCTION

The glass ceiling metaphor is frequently used to describe the obstacles and barriers in front of women seeking promotions to the top levels of organizations.^1–2^ There is strong evidence of the under-representation of women in leadership positions in countries such as Australia,^3^ China,^4^ France,^5^ and the United States.^6^

There is a strong need for ongoing investigations into the causes and consequences of glass ceilings, especially in corporate organizations. Research on the glass ceiling shows that women may encounter obstacles in pursuing high-level management positions. Hymowitz and Schellhardt introduced the term glass ceiling in their 1986 Wall Street Journal article.^7^ Although they were the first to use the metaphor, they were not the first to write about the challenges women faced as they attempted their climb up the corporate ladder to senior-level positions. Hymowitz and Schellhardt remarked that even women who successfully climbed the corporate ladder would eventually crash into an invisible barrier. Although high-level positions appeared to be within women's reach, they could not crack the glass ceiling. Since these remarks were made, others have continued the effort to better understand the antecedents of the phenomenon through research, which has led to the development of theories attempting to explain the phenomenon.^7^

Prosthetics and orthotics are established disciplines in health science and are often practiced together as they have many commonalities from clinical, technical, and therapy perspectives. Prosthetic and orthotic devices are usually part of the secondary/tertiary care, habilitation, and rehabilitation programs. The number of female prosthetists and orthotists has risen worldwide. In the United States only, according to the American Orthotic & Prosthetic Association (AOPA), in 2014, 20% of practitioners who had registered with the American Board for Certification in Orthotics, Prosthetics and Pedorthics (ABC) were women, a 748% increase since 1994; ABC also estimates women and men now enter O&P in about a 1:1 ratio. Among professional members of the American Society for Biomechanics (ASB) who report their gender, 31% are women, as are 42% of student members (2016 data).^8^

Women have proved their competence as successful senior-level leaders. Despite this, there is still a striking under-representation of female prosthetists and orthotists in leadership positions as managers and business owners.^9^ It is unknown what female graduates expect from their future careers, and if those expectations are realistic, especially when it comes to managerial positions. Also, it is not known what role cultural context plays in success expectations and professional promotions among females, and other nontraditional prosthetists and orthotists.

Research in other fields, such as medicine, banking, and sports management, emphasize gender differences as the major reason for gender inequality in leadership.^10–13^ The glass ceiling is supported by conscious and unconscious gender stereotypes and biases, a lack of policies that support work-life balance, a lack of mentors or role models for women interested in high-level career advancement, and a paucity of networks that can open doors to women. Nothing is known about other diversity barriers in prosthetics and orthotics, such as age, place of origin or education, or language proficiency. The field is small and internationally connected. Anecdotally, there is a sense that the location of education is a significant barrier to entering the field of prosthetics and orthotics. Therefore, this study is the first to develop pilot information on women's beliefs about glass ceilings among female prosthetists and orthotists.

## METHODOLOGY

Ethics approval was obtained from the British Columbia institute of technology (BCIT) Research Ethics Board (2021-05). The first part of this survey study measured the demographics: age, marital status, number of children, years of work experience in P&O, qualification, and country of work (**Table 1**). Both male and females participants could fill in this part.

**Table 1:** Summary statistics of four sets of beliefs about the glass ceiling based on the Career Pathway Survey.

Factors		Denial Mean (SD)	Resilience Mean (SD)	Resignation Mean (SD)	Acceptance Mean (SD)
**Age (year)**	18–35	**29.3 (11.5)** ^a^	40.0 (14.4)	55.5 (6.6)	23.2 (6.9)
	36–45	33.0 (12.93)	36.5 (10.7)	56.3 (7.1)	24.9 (5.5)
	46+	**38.3 (14.3)** ^a^	35.2 (13.0)	55.6 (12.6)	26.6 (7.02)
**Marital Status**	Married	33.6 (13.4)	36.9 (12.2)	56.5 (8.5)	24.8 (6.5)
	Single	29.5 (11.5)	40.0 (14.6)	54.7 (7.3)	23.7 (6.7)
**Number of Children**	None	**29.1 (11.3)** ^b^	39.3 (14.0)	54.3 (8.9)	22.4 (6.1)
	At least one child	**34.6 (13.6)** ^b^	36.0 (11.9)	56.8 (7.6)	25.7 (6.1)
**Years of Experience**	0–5	29.2 (11.6)	39.9 (15.0)	54.6 (7.4)	22.3 (7.7)
	6–10	29.4 (11.1)	39.9 (13.8)	56.0 (5.01)	25.3 (5.5)
	11–15	35.7 (13.7)	35.2 (11.4)	56.5 (7.7)	23.6 (5.8)
	16–25	31.4 (13.2)	39.7 (11.1)	55.6 (11.5)	26.1 (6.7)
	>26	40.0 (14.0)	31.6 (11.8)	57.1 (9.5)	26.4 (5.7)
**Country of Work**	Europe	30.4 (7.4)	41.9 (9.1)	56.2 (6.3)	24.1 (6.4)
	Australia	30.3 (10.1)	34.9 (12.4)	54.5 (7.6)	24.0 (5.4)
	North America	31.7 (13.8)	37.3 (14.1)	55.5 (9.0)	24.0 (6.9)
**Qualification**	1–4 Semesters	34.2 (14.0)	36.6 (13.5)	56.9 (7.9)	25.3 (6.2)
	Bachelor	33.7 (10.6)	40.9 (11.6)	55.5 (9.0)	25.0 (6.3)
	Master	28.1 (14.9)	35.8 (13.3)	55.5 (6.9)	21.3 (6.7)

a Shows a statistically significant difference in mean denial scores between age groups, 18-35 and 46+ at the 0.05 level of significance.

b Shows a statistically significant difference in mean denial scores between the number of children groups, no children and at least one child at the 0.05 level of significance.

The second part involved the Career Pathways Survey (CPS) adopted from Smith et al.^14,15^ This part was only asked to be answered by female participants. The CPS is a multi-factorial instrument which quantitatively assesses four sets of beliefs about glass ceilings: *Denial, Resilience, Acceptance, and Resignation*. The CPS provides scores for four groups of beliefs about glass ceilings.

- ***Denial:*** Denial is defined as the belief that men and women face the same issues and problems in seeking leadership. Examples of items in the CPS assessing Denial are: “*Women have reached the top in all areas of business and politics*”, and “*Women starting careers today will face sexist barriers*” (reverse scored).
- ***Resilience:*** Resilience is defined as the belief that women are able to break glass ceilings. Examples of this factor are: “*The more women seek senior positions, the easier it will be for those who follow”*, and “*Women are capable of making critical leadership decisions*”.
- ***Acceptance:*** Acceptance is the belief that women prefer other life goals, such as family involvement, over developing a career. Therefore, Acceptance is summed up as a pro-family/anti-career advancement set of beliefs. Examples of items in the CPS assessing acceptance are: “*Women reject the need to work incredibly long hours*”, and “*Women are less concerned about promotions than men are*”.^15^
- ***Resignation:*** Resignation is the belief that women suffer many more negative consequences than men when pursuing career advancement and thus, there are overwhelming reasons for women not attempting to break glass ceilings. Two CPS items measuring this factor are: “*Women are more likely to be hurt than men when they take big risks necessary for corporate success*”, and “*Jealousy from coworkers prevents women from seeking promotions*”.

The 38 items of the CPS represent beliefs about a wide variety of variables that considerable research has shown to be linked to women's career advancement.^7,16^ For example, the CPS items refer to issues such as role models, lack of promotion opportunities for women, sexist barriers in organizations, successful organizations wanting talented women leaders, work-family compromises, benefits of higher education, networking, jealousy from female colleagues after promotions, support from mentors, and sexual harassment. Participants rated their level of agreement with 38 statements on a scale from 1 (strongly disagree) to 7 (strongly agree). Individual factor scores are calculated by the mean score of the relevant items.

### Data collection

All individuals (male and female) with a professional qualification in prosthetics and/or orthotics that was valid in the region in which they worked were eligible to participate in the survey. Subjects were recruited by posting on the OandP List-Serve and distribution of an invitation to participate by the Orthotics Prosthetics Canada (OPC), British Association of Prosthetists & Orthotists (BAPO), American Orthotic & Prosthetic Association (AOPA), and International Society for Prosthetics and Orthotics (ISPO). Moreover, persons receiving the invitation were asked to forward the invitation to Prosthetists and Orthotists in their networks (snowball sampling method). The link in the invitation directed interested persons to an explanatory letter with information on the study purpose, inclusion criteria, confidentiality, risk, and consent, along with a link to a SurveyMonkey that enabled the anonymous submission of responses to the questionnaire (**Appendix A and B**).

### Statistical Analysis

Statistical analyses were performed using SAS 9.4M7 (SAS Institute, Cary, North Carolina). One-way analysis of variance (ANOVA) was used to test for differences in mean scale response variables (Denial, Resilience, Acceptance, and Resignation) between levels of categorical demographic variables. A separate one-way ANOVA was conducted for each of these scale responses. Post hoc tests using a Tukey-Kramer adjustment were performed to locate differences in mean responses between pairs of levels of demographic variables. The standard model assumptions regarding the residuals were verified. The residuals were approximately normally distributed random variables centered about zero with constant variance. In cases where outliers were detected, the analysis was re-run removing the outlier, but none of the final model conclusions changed, so the outliers remained in the model. Sample size was determined using GPower, with an expected effect size of 0.8, alpha level of 0.05, and power of 95%.

## RESULTS

110 female participants completed the survey. The survey was opened in April 2021 and remained active for six months. Some participants did not answer all the questions and therefore were not included in the data analysis to avoid potential issues with model estimation. The participants were mainly from North America (n = 56), Europe (n = 18) and Australia (n = 21) followed by Asia (n = 9) and Africa (n = 6). **Table 1** shows the summary statistics for each set of beliefs broken down by each level of the demographic variable. The responses from Asia and Africa were excluded from the analysis due to the number of participants needed for model estimation.

The one-way ANOVA revealed a statistically significant difference in mean denial scores between age groups (F2,107 = 3.21, p = 0.04). A post-hoc Tukey-Kramer test for multiple comparisons found that the mean value of denial scores was statistically significantly (p-value = 0.03) higher in the 46+ age group (mean = 38.3) than in the 18–35 age group (mean = 29.3).

**Table 2** represents results of one-way analysis of variance (ANOVA) for differences in mean scale response variables (Denial, Resilience, Acceptance, and Resignation) between levels of categorical demographic variables.

**Table 2.** One-way analysis of variance (ANOVA) for differences in mean scale response variables (Denial, Resilience, Acceptance, and Resignation).

Pearson Correlation Coefficients Prob > |r| under H0: Rho=0 Number of Observations					
	Denial	Resignation	Resilience	Acceptance
**Denial**	1.00	−0.41	0.01	0.24
	–	<.0001	0.95	0.02
	99.00	99.00	97.00	96.00
**Resignation**	−0.41	1.00	−0.07	0.16
	<.0001	–	0.49	0.11
	99.00	99.00	97.00	96.00
**Resilience**	0.01	−0.07	1.00	0.08
	0.95	0.49	–	0.43
	97.00	97.00	97.00	96.00
**Acceptance**	0.24	0.16	0.08	1.00
	0.02	0.11	0.43	–
	96.00	96.00	96.00	96.00

The one-way ANOVA revealed a statistically significant difference in mean denial scores between the number of children groups (F1,88 = 4.47, p = 0.04). A post hoc Tukey-Kramer test found that the mean value of denial scores was statistically significantly (p-value = 0.04) higher in participants with one child (mean = 34.6) compared to those with no children (mean = 29.1). A borderline statistically significant difference was seen in mean acceptance scores between academic qualification groups (F2,87 = 2.87 p = 0.06). The mean acceptance scores were higher for employees with 1–4 semesters of education (mean = 34.2) compared to employees with master's degrees (mean = 28.1).

The type III tests of fixed effects from a multiple regression model in **Table 3** show that salary is a statistically significant predictor of mean denial scores with p-values of 0.02. **Table 3** shows there is a statistically significant difference (p-value = 0.02) in mean denial scores between subjects with salary >40k (mean = 34.6) and salary <40k (mean = 27.3). Resilience was predicted by both salary >40k and having no children. Resilience scores were significantly higher for people with salary >40k compared to salary <40k (F1, 92 = 5.6, p = 0.02). The lack of children in an individual's demographic category significantly predicted increased resilience scores (F1, 92 = 3.7, p = 0.04).

**Table 3:** Results of multiple regression model.

	DF	Type III SS	Mean Square	F Value	Pr > F
**Salary**	1	567.33	567.33	5.56	0.02
**Education Cat**	2	396.63	198.31	1.94	0.16
**Age**	2	46.92	23.46	0.23	0.80
**Country of Work**	2	222.23	111.12	1.09	0.35
**Years of Experience**	4	531.57	132.89	1.30	0.29
**Number of Children**	1	166.17	166.17	1.63	0.20

A weak positive correlation was detected between number of children and denial (r = 0.27, p < 0.01) and resilience scores (r = 0.23, p-value = 0.02) (**Table 4**).

**Table 4:** Results of Pearson correlation for number of children and hours of work in relation to four sets of beliefs about glass ceilings.

Pear Correlation Prob > |r| under H0: Rho=0 Number of Observations				
	Denial	Resignation	Resilience	Acceptance
**Number of Children**	0.27	−0.16	0.23	0.15
	0.01	0.11	0.02	0.14
	100.00	100.00	98.00	–
**Hours Work in Week**	0.10	0.00	0.04	−0.13
	0.32	0.99	0.71	0.17
	109.00	109.00	107.00	106.00

## DISCUSSION

This study investigated the perceptions and beliefs of female prosthetists and orthotists regarding the glass ceiling and professional advancement. The findings indicate a complex interplay of factors that contribute to barriers in career progression for women in the field, including gender, family status, and educational background. These results are consistent with studies in other fields that have found that women often encounter significant obstacles in advancing to senior leadership positions.^2^ Similar to other professions, the findings of this study is an empirical evidence that the glass ceiling phenomenon still exists for female prosthetists & orthotists.^17–19^ One major finding was the difference in denial scores among age groups, with older participants exhibiting more denial about the existence of gender-based barriers than younger participants. This suggests that, over time, women may become more aware of the glass ceiling or, conversely, become more accepting of its limitations as they progress in their careers.

Furthermore, the study showed that women with one child were more likely to deny the existence of gender-based barriers compared to those without children. This might be due to the demands of balancing career and family life, which may lead to a pragmatic acceptance of organizational limitations.^20^ In contrast, women without children were found to have higher resilience scores, which may reflect fewer family-related barriers to professional advancement. This is consistent with the finding that women with fewer family commitments are better able to dedicate time and energy to career advancement.^17^ In some professions, it is possible to work from home, which provides an opportunity to take care of children.^21^ This is barely possible for orthotist & prosthetist clinician and technicians due to responsibilities of direct in-person patient care and manufacturing requirements that should be done in a prosthetic and orthotic workshop.

Educational background also played a significant role in the beliefs about glass ceilings. Participants with lower levels of education (1–4 semesters) reported higher acceptance of the idea that women prefer family life over career advancement. This suggests that education, especially higher education, may increase awareness of career opportunities and career advancement processes.

Salary was also identified as a significant predictor of denial, with individuals earning more than $40,000 reporting higher denial scores. This suggests that higher earnings may lead to a greater sense of professional success and thus reduce the perceived impact of gender-related barriers to career advancement.

These findings are valuable for understanding the factors that contribute to the under-representation of women in leadership roles within prosthetics and orthotics, as they point to a combination of personal, professional, and cultural influences that impact career progression. Furthermore, they underscore the importance of developing policies and programs that support work-life balance, professional development, and mentorship to help break the glass ceiling in this field.^15,22^

### Limitations

To our knowledge, there is no other similar research work done and published on this topic in P&O so we couldn't make comparisons across the literature. Our initial intention was to do sampling worldwide but during the time that survey was active, we got mainly participants from North America, Europe and Australia, while only few from other regions so we could not include those participants in the data analysis. The literature shows that glass ceiling may be experienced more in low- and middle-income countries; therefore, the future study will seek sampling methods that would increase participation from those areas.

In this study, we only asked female clinicians to answer the glass ceiling survey while it is worthwhile to know how male clinicians perceive the glass ceiling. Both males and females may experience glass ceilings and therefore we will consider including both genders in the study in future study. Another limitation was that not all the participants answered every section and/or question on the survey, which made it difficult to come to conclusions or study the relationship between all variables.

## CONCLUSION

Since the latter half of the 20th century, women have made great strides in increasing their representation in the work force. However, a considerable gap remains in achievement of leadership positions across fields such as healthcare. Thus, the bewildering glass ceiling remains intact and, at times, seemingly invulnerable. We propose that glass ceiling beliefs can lift or diminish desires to be promoted. These beliefs may lead to career pathway choices and longlasting behaviors within organizations. Women who express an ambition to become part of upper management, could gain insights by analyzing their levels of Resilience and Denial. High Acceptance scores could help identify women with little or no ambition to be promoted, yet would benefit from professional development whilst maintaining their level in the organization. Finally, feedback from CPS testing might also facilitate women gaining greater awareness of the possible causes for their subjective success in organizations. Those women who score high on Resignation could benefit from training and development courses that help them examine the validity of their negative thoughts about women seeking promotions. However, if it is found that an organization's structure and actions do indeed lead to Resignation, major changes will be needed before women in that organization can dismiss their negative beliefs.

## DECLARATION OF CONFLICTING INTERESTS

The authors report no conflict of interest.

## AUTHORS CONTRIBUTION

**Arezoo Eshraghi:** Conceptualization, Ethics Application, Methodology, Data Collection and Analysis, Writing/Revising the Manuscript, Final Manuscript Approval.

**Zahra Safaeepour:** Conceptualization, Ethics Application, Methodology, Data Collection and Analysis, Writing/Revising the Manuscript, Final Manuscript Approval.

## SOURCES OF SUPPORT

British Columbia Institute of Technology (BCIT) MAKE+.

## APPENDIX

### Appendix A: Explanatory Letter with Survey (February 2, 2021)

**Baseline Survey: Glass ceilings in prosthetics & orthotics: Perceptions, opportunities and strategies for moving forward**

Principle Investigator: Silvia Raschke, PhD, BCIT MAKE+ Tel: 604-412-7597 E-Mail: Silvia_Raschke@bcit.ca

Co-Investigator:  Zahra Safaeepour, PhD University of South Carolina Upstate.

Co-Investigator:  Arezoo Eshraghi, PhD, CP (c) West Park Healthcare Centre.

The purpose of this project is to examine perceptions and beliefs that contribute to the glass ceiling effect for persons choosing a career in prosthetics and orthotics. Factors such as gender, age and career aspirations will be explored. There will be no compensation provided for participating in the survey.

This study will provide baseline survey data to take an objective snapshot of the prosthetics and orthotics profession in 2021. The researchers intend to apply for grant funding to explore this topic more deeply with the goal of developing strategies, recommendations and tools for engaging and recruiting persons not traditionally considering prosthetics and orthotics as a career, with a focus on females. Results will also be presented at a conference(s) and will be submitted for publication in a peer-reviewed journal.

The survey consists of two parts:

- Part A is for all participants and should take between 10 and 15 minutes to complete.

Part B is an additional set of questions for all participants identifying as female and will take approximately 15 additional minutes to complete.

**The survey is open to all persons who hold a recognized international qualification in prosthetics and/or orthotics (e.g Bachelor's Degree, Certification, Licensure, Meister, etc.) and who have reading and writing comprehension in English It is not necessary for you to be qualified to practice in your country of residence.**

To keep your identity confidential, no personally identifiable information will be collected or stored during this research project (i.e. you will not be identifiable in any reports, publications or presentations resulting from this study). All data and comments will be collapsed into a summary document. Please note that researchers may be required to make the survey data publicly available at the time of publication. You should be aware that once data is made publicly available you will not be able to withdraw any comments. If, at any time during the survey you wish to withdraw from the survey you can do so at any point by simply closing the browser.

There is minimal anticipated risk to participating in this survey. If you would like to have a summary of the results of the survey or if you have any questions, or concerns, regarding this survey or your participation you can email me directly at: Silvia_Raschke@bcit. If there are any further concerns you may contact the Research Ethics Board at research_ethics@bcit.ca.

### Appendix B: Survey Questions

**Project: Baseline Survey: Glass ceilings in prosthetics & orthotics: Perceptions, opportunities and strategies for moving forward**

#### PART A Survey Questions (for all respondents)

##### Professional History

1- Highest Level of General Education (Check one):
  _____Apprenticeship
  _____1 to 2 Semester Certificate
  _____2 to 4 Semester Diploma/Certificate
  _____Bachelor's Degree
  _____Master's Degree
  _____PhD
  _____Other: (please specify) ____________________
2- Country in which you received your highest level of education: ________
3- Country(ies) in which you received your Prosthetics and Orthotics education:
  Note: If you have more than one certification and/or degree please note each completed Certificate/Diploma and/or Degree and, in brackets next to it, note the country in which it was obtained. (e.g. Master's Degree (UK))
  **Table:**
  1. First Certificate/Diploma/Degree (country obtained)
  2. Second Certificate/Diploma/Degree (country obtained)
  3. Third Certificate/Diploma/Degree (country obtained)
  4. Fourth Certificate/Diploma/Degree (country obtained)
4- Highest Level of Prosthetics and Orthotics Specific Education:
  Check one:
  _____Apprenticeship
  _____1 to 2 Semester Certificate
  _____2 to 4 Semester Diploma/Certificate
  _____Bachelor's Degree
  _____Master's Degree
  _____PhD
  _____Other: (please specify) ____________________
5- Country in which you work : _________________
6- Country in which you live/are a resident of: _______________
7- If you are seeking work in a country different from where you currently work and/or live, what is the country in which you are seeking work:
8- Professional Qualification(s) held in Prosthetics and Orthotics (over and above general education degree):
  (Choose all that apply)
  ______Certified Orthotist,
  ______Certified Prosthetist,
  ______Licensed Orthotist,
  ______Licensed Prosthetist,
  ______Certified Prosthetist Orthotist,
  ______ Meister.
  ______Registered Prosthetic Technician,
  ______Registered Orthotic Technician
  ______Other (please specify):
9- Is this qualification required for you to work in the country of your education? _____ Yes_____ No
10- Is this qualification required for you to work in your country of residence?_____ Yes _____ No
11- Is this qualification recognized in the country in which you are aspiring to work in? _____ Yes _____ No _____ Not Applicable
12 - How many years have you worked in the field of Prosthetics and Orthotics? ________
13 - What is your current employment status in the field of Prosthetics and Orthotics?
  ____Employed full-time
  ____Employed part-time
  ____Self-employed
  ____Unemployed, seeking opportunities
  ____Unemployed, not seeking opportunities
  ____Retired
  ____Other: _________
14 - How many hours per week are you currently working in the field of Prosthetics and Orthotics? _____
15 - Would having the ability to have a permanent part-time position be of interest to you at any point in your career? (yes/no)
16 - Have you ever sought a permanent part-time position at any point in your career? (yes/no)
17 - If yes, were you able to find a permanent part-time position? (yes/no)

##### General Exploratory Survey

18 - This question asks you to consider your personal professional aspirations as a Prosthetics and Orthotics practitioner.
  E.g. My ultimate career goal is to: ^*^
  - be a dual qualified prosthetist orthotist
  - be leading prosthetic and orthotic research
  - be working in a public hospital or clinic
  - own my own prosthetics/orthotics business
  - be managing a hospital department
  - be working for a private practice
  - be working clinically part time and will spend the remainder of my time doing (x).
  - be carrying out prosthetic and orthotic research
  - Other ___________________
  ^*^Note: the examples given above have been randomized (other than the last item ‘other’) in order to mitigate order bias.
  In each box below, identify up to 3 professional aspirations you hope to achieve in the field of prosthetics and orthotics field (max. 30 words, 10 per box)
  **Table:**
  1-
  2-
  3-
19 - What is the highest professional level you anticipate you will have achieved in your career as a certified prosthetics/orthotics practitioner? (pick one)^*^
  ____ Employee
  ____ Researcher
  ____ Will have left the profession
  ____ Manager
  ____ Business owner
  ____ Other: ___________________________(please provide details)
  ^*^Note: the examples given above have been randomized (other than the last item ‘other’) in order to mitigate order bias 20 - In the region where you live what is the **most common** business model in P&O? (pick one)^*^
  ______ Private Clinic with multiple employees
  ______ Prosthetic and Orthotic Services are provided by a Non Governmental Organization (NGO)
  ______ Public Service Hospital/Clinic
  ______ Small Family Businesses
  ______ Other ___________________________(please describe on line provided)
  ^*^Note: the examples given above have been randomized (other than the last item ‘other’) in order to mitigate order bias
21 - In the region where you live what is the **second most common** business model in P&O? (pick one)^*^
  ______ Prosthetic and Orthotic Services are provided by a Non Governmental Organization (NGO)
  ______ Small Family Businesses
  ______ Private Clinic with multiple employees
  ______ Public Service Hospital/Clinic
  ______ Other ___________________________(please describe on line provided)
  ^*^Note: the examples given above have been randomized (other than the last item ‘other’) in order to mitigate order bias 22 - Describe what success in your chosen career of Prosthetics and Orthotics looks like for you. (max. 100 words)
  **Table:**
23 - Do you see any barriers to your achieving personal success in prosthetics and orthotics, as you have described above? _ Yes_ No
  If yes, please list those barriers in the box below (max 25 words).
  **Table:**
24 - Describe what the glass ceiling looks like in prosthetics and orthotics, from your perspective, even if you have not experienced it. If you
  Next we will explore what a Glass Ceiling looks like in the fields of Prosthetics and Orthotics.
  A Glass Ceiling is described as:
  “Invisible artificial barriers (sometimes generated by management) that can limit the career advancement of employees, particularly women and members of minority groups. The expectations and aspirations of all staff within an organization should be met equally. While standards of practice in this area are laid down by law in many countries, they are not always observed.”
  From: Oxford Reference: https://www.oxfordreference.com/view/10.1093/oi/authority.20110803095854441
  For the purpose of this General Exploratory Survey component, this question applies to ALL PERSONS who could experience ‘invisible artificial barriers that limited <their> career advancement” for any range of reasons that include but are not limited to: gender, age, country of origin or education.
  do not believe a glass ceiling exists, please note that.
25 - Have you ever experienced a glass ceiling effect in the course of your career?__ Yes __ No
26 - If yes, describe your glass ceiling experience in the box below. (max. 150 words)
  **Table:**
27 - Which identifying factor do you believe was the root of your experience with the glass ceiling described above?^*^ Check all that apply:
  _____ race
  _____ age
  _____ disability
  _____ gender
  _____ country of education
  _____ cultural background
  _____ language
  _____ religion
  _____ country of origin
  _____ other __________________________(describe)
  ^*^Note: the examples given above have been randomized (other than the last item ‘other’) in order to mitigate order bias
28 - If you trained in a country different from the one you now live and subsequently sought employment in Prosthetics and Orthotics, did you face any barriers on the path to employment in Prosthetics and Orthotics in the country in which you now live/work in? _ Yes _ No
29 - If yes, please list up those barriers in the box below (max 40 words) i.E.g. ^*^ difficulty in: finding work, having a credential recognized, adapting to clinical and technical practices in country you are now working in, adapting to workplace culture, language difficulties, etc.
  ^*^Note: the examples given above have been randomized (other than the last item ‘other’) in order to mitigate order bias
  **Table:**

##### 2- Demographic Details

30 - What is your age?
  _____18-25
  _____26-35
  _____36-45
  _____46-55
  _____56-65
  _____65+
31- Country of birth: (pull down menu)
32- Which of the following best represents your racial and/or heritage? (non-mandatory question)
  Choose all that apply.
  ___Hispanic or Latino
  ___East Asian
  ___White or Caucasian
  ___Middle Eastern
  ___Native Hawaiian or Pacific Islander
  ___Mixed
  ___South Asian
  ___Asian
  ___First Nations or Indigenous
  ___Black or African
  ___Other preferred identifier: _____________
  Please print your specific ethnicities in the space below, if you wish.
  (Examples of ethnicities include (for example): German, Korean, Mexican American, Navajo Nation, Samoan, Puerto Rican, Southerner (American), Chinese, etc. Note, you may report more than one group.)
  Ethnicity(s) _________________________________
  ^*^Note: the above have been randomized (other than the last two items) in order to mitigate order bias.
  ^**^Note: There are numerous potential lists that could be used for this question. We have used a list that combines what is used by researchers in US Veteran's Association research applications and Holland Bloorview Hospital (Toronto). We anticipate most respondents will come from US/US Territories and Canada so the categories will be familiar to them. In addition the simplicity of this list will be understandable to persons from outside this geographic area who may also answer (through posting/distribution via the International Society for Prosthetics and Orthotics network).
33- Marital status:
  ___Single (never married)
  ___Married, or in a domestic partnership
  ___Widowed
  ___Divorced
  ___Separated
34- Number of children: ______
35- The gender you identify as is:^*^
  ____Female
  ____Male
  ____Other (provide gender identity, if you wish, in the space provided): ___________
^*^Note: the examples given above have been randomized (other than the last item ‘other’) in order to mitigate order bias
  Note: Only persons choosing the Female option above will proceed to Part B.
  All others will be send to a page with the submit button.
  The follow reminder will placed above the Submit button:
  *Clicking on the Submit button below will submit your data to the survey, after which it can no longer be retrieved or changed.*
  *If you wish to withdraw from the study, you can still do so at this point by simply closing the browser.*
  After clicking the submit button they will be taken to a page thanking them for participating them in the survey.

##### PART B Survey Questions (for respondents identifying as female)

###### Career Pathways Survey

**Please consider each of the statements below and rank them from 1 (strongly disagree) to 7 (strongly agree)**

**Table:**

Women starting careers today will face sexist barriers.						
(strongly disagree)		(Neutral)			(strongly agree)	
1	2	3	4	5	6	7
Women and men have to overcome the same problems at the workplace.						
(strongly disagree)		(Neutral)			(strongly agree)	
1	2	3	4	5	6	7
It will take decades for women to reach equality with men in high level management positions.						
(strongly disagree)		(Neutral)			(strongly agree)	
1	2	3	4	5		7
Even women with many skills and qualifications fail to be recognized for promotions.						
(strongly disagree)		(Neutral)			(strongly agree)	
1	2	3	4	5	6	7
Women have reached the top in all areas of business and politics.						
(strongly disagree)		(Neutral)			(strongly agree)	
1	2	3	4	5	6	7
Women face no barriers to promotions in most organizations.						
(strongly disagree)		(Neutral)			(strongly agree)	
1	2	3	4	5	6	7
Women leaders are seldom given full credit for their successes.						
(strongly disagree)		(Neutral)			(strongly agree)	
1	2	3	4	5	6	7
Women in senior positions face frequent putdowns of being too soft or too hard.						
(strongly disagree)		(Neutral)			(strongly agree)	
1	2	3	4	5	6	7
Women who have a strong commitment to their careers can go right to the top.						
(strongly disagree)		(Neutral)			(strongly agree)	
1	2	3	4	5	6	7
Talented women are able to overcome sexist discrimination.						
(strongly disagree)		(Neutral)			(strongly agree)	
1	2	3	4	5	6	7
Women executives are very uncomfortable when they have to criticize members of their teams.						
(strongly disagree)		(Neutral)			(strongly agree)	
1	2	3	4	5	6	7
Women leaders suffer more emotional pain than men when there is a crisis within their teams.						
(strongly disagree)		(Neutral)			(strongly agree)	
1	2	3	4	5	6	7
Being in the limelight creates many problems for women.						
(strongly disagree)		(Neutral)			(strongly agree)	
1	2	3	4	5	6	7
Women are more likely to be hurt than men when they take big risks necessary for corporate success.						
(strongly disagree)		(Neutral)			(strongly agree)	
1	2	3	4	5	6	7
Women believe they have to make too many compromises to gain highly paid positions.						
(strongly disagree)		(Neutral)			(strongly agree)	
1	2	3	4	5	6	7
Jealousy from co-workers prevents women from seeking promotions.						
(strongly disagree)		(Neutral)			(strongly agree)	
1	2	3	4	5	6	7
Even very successful women can quickly lose their confidence.						
(strongly disagree)		(Neutral)			(strongly agree)	
1	2	3	4	5	6	7
Women know that work does not provide the best source of happiness in life.						
(strongly disagree)		(Neutral)			(strongly agree)	
1	2	3	4	5	6	7
If women achieve promotions, they might be accused of offering sexual favours.						
(strongly disagree)		(Neutral)			(strongly agree)	
1	2	3	4	5	6	7
Smart women avoid careers that involve intense competition with colleagues.						
(strongly disagree)		(Neutral)			(strongly agree)	
1	2	3	4	5	6	7
The more women seek senior positions, the easier it will be for those who follow.						
(strongly disagree)		(Neutral)			(strongly agree)	
1	2	3	4	5	6	7
Higher education qualifications will help women overcome discrimination.						
(strongly disagree)		(Neutral)			(strongly agree)	
1	2	3	4	5	6	7
Women have the strength to overcome discrimination.						
(strongly disagree)		(Neutral)			(strongly agree)	
1	2	3	4	5	6	7
When women are given opportunities to lead, they do effective jobs.						
(strongly disagree)		(Neutral)			(strongly agree)	
1	2	3	4	5	6	7
Daughters of successful mothers are inspired to overcome sexist hurdles.						
(strongly disagree)		(Neutral)			(strongly agree)	
1	2	3	4	5	6	7
Women are capable of making critical leadership decisions.						
(strongly disagree)		(Neutral)			(strongly agree)	
1	2	3	4	5	6	7
A supportive spouse/partner or close friend makes it easier for a woman to achieve success in her career.						
(strongly disagree)		(Neutral)			(strongly agree)	
1	2	3	4	5	6	7
Successful organizations seek and want to retain talented female staff.						
(strongly disagree)		(Neutral)			(strongly agree)	
1	2	3	4	5	6	7
The support of a mentor greatly increases the success of a woman in any organization.						
(strongly disagree)		(Neutral)			(strongly agree)	
1	2	3	4	5	6	7
Women's nurturing skills help them to be successful leaders.						
(strongly disagree)		(Neutral)			(strongly agree)	
1	2	3	4	5	6	7
Networking is a smart way for women to increase the chances of career success.						
(strongly disagree)		(Neutral)			(strongly agree)	
1	2	3	4	5	6	7
Women are just as ambitious in their careers as men.						
(strongly disagree)		(Neutral)			(strongly agree)	
1	2	3	4	5	6	7
Women have the same desire for power as men do.						
(strongly disagree)		(Neutral)			(strongly agree)	
1	2	3	4	5	6	7
Motherhood is more important to most women than career development.						
(strongly disagree)		(Neutral)			(strongly agree)	
1	2	3	4	5	6	7
Women are less concerned about promotions than men are.						
(strongly disagree)		(Neutral)			(strongly agree)	
1	2	3	4	5	6	7
Women prefer a balanced life more than gaining highly paid careers.						
(strongly disagree)		(Neutral)			(strongly agree)	
1	2	3	4	5	6	7
Women reject the need to work incredibly long hours.						
(strongly disagree)		(Neutral)			(strongly agree)	
1	2	3	4	5	6	7
Women commonly reject career advancement as they are keener to maintain a role raising children.						
(strongly disagree)		(Neutral)			(strongly agree)	
1	2	3	4	5	6	7

**For those completing Part B: On completion of Part B the respondent will be taken to a Submit button that looks the same as what the respondents who did Part A only looks like:**

The follow reminder will placed above the Submit button:

*Clicking on the Submit button below will submit your data to the survey, after which it can no longer be retrieved or changed.*

*If you wish to withdraw from the study, you can still do so at this point by simply closing the browser.*

After clicking the submit button they will be taken to a page thanking them for participating them in the survey.
